# The Impact of COVID-19 on the Health and Experience of the Carers of Older Family Members Living with Dementia: An Italian–Hungarian Comparative Study

**DOI:** 10.3390/ijerph19095329

**Published:** 2022-04-27

**Authors:** László Árpád Kostyál, Zsuzsa Széman, Virág Erzsébet Almási, Paolo Fabbietti, Sabrina Quattrini, Marco Socci, Cristina Gagliardi

**Affiliations:** 1 Institute of Mental Health, Semmelweis University, P.O. Box 2, 1428 Budapest, Hungary; kostyal.arpad@public.semmelweis-univ.hu (L.Á.K.); szeman.zsuzsanna@public.semmelweis-univ.hu (Z.S.); 2 Independent Researcher, 1191 Budapest, Hungary; virag.almasi@gmail.com; 3 Unit of Geriatric Pharmacoepidemiology and Biostatistics, IRCCS INRCA-National Institute of Health and Science on Ageing, Via Santa Margherita 5, 60124 Ancona, Italy; p.fabbietti@inrca.it; 4 Centre for Socio-Economic Research on Ageing, IRCCS INRCA-National Institute of Health and Science on Ageing, Via Santa Margherita 5, 60124 Ancona, Italy; s.quattrini@inrca.it (S.Q.); c.gagliardi@inrca.it (C.G.)

**Keywords:** COVID-19, family carers/caregivers, older people, dementia, care, comparative study, support services, care needs

## Abstract

This quantitative study (*n* = 370) compares the pandemic-related experiences of the family carers of older people living with dementia during the first wave of the pandemic, in two countries with different care regimes: Italy (Mediterranean) and Hungary (Eastern European). It aims at answering the following research questions: (1) How did the pandemic affect the subjective health of carers, and what were their experiences with care-related worries and workload? (2) What factors significantly predicted negative changes in these experiences? (3) What were carers’ main difficulties during the first pandemic wave? Results have shown that carers in both samples reported a worsening in mental health (Italy/Hungary: M = 2.25/2.55, SD = 0.93/0.99), and Italian carers also in general health (M = 2.54, SD = 0.98) (on a scale of 1 to 5, with values under “3” representing deterioration). Carers in both samples experienced high worry levels (Italy/Hungary: M = 4.2/3.7, SD = 0.93/0.89) and feeling overwhelmed with care tasks (M = 3.2/3.7, SD = 1.3/1.3) (on a scale of 1 to 5, higher values representing higher worry/work overload). In regression models, all of the above negative experiences were predicted by a combination of factors. Two of these factors stood out in importance due to being a predictor of more than one type of negative experience: a decline in the carer–care receiver relationship, predicting work overload, as well as general and mental health deterioration and being the child of the care receiver, predicting both high worry and subjective work overload. The top five encountered problems were the unavailability of medical and social care, difficulties with shopping (medicine included), restricted freedom, isolation, and anxiety.

## 1. Introduction

The total number of people living with dementia is projected to reach 82 million in 2030 and 152 million in 2050. The estimated proportion of the population aged 60 and over with dementia at any given time is between five and eight per cent [1]. Dementia ranges from mild (early-stage) to severe (late-stage), and the probability of a more severe stage increases substantially with age. The more advanced the stage of dementia, the more care, support, and attention the ill person requires. The distribution of costs of the care of dementia underpins the important role of informal caregivers, including family carers. Direct medical care costs account for 20% of global dementia costs. The direct social sector costs are 40% and the informal care costs are 40% [2]. Carers may experience a heavy emotional, social, financial, and physical burden [3,4]. The comparative study of Ory et al. [5] documented that dementia caregivers spend significantly more hours per week providing care compared to non-dementia caregivers.

The set of responsibilities that comes with care work can turn into a burden that puts a strain on the psychophysical well-being of the caregiver. The behavioural and psychological symptoms associated with dementia make the management of the person living with this illness a complex task, which can be especially trying for the caregiver [6,7,8]. The caregiver is often alone and has little support from other people to share these tasks with, resulting in feeling lonely [9]. Objective stressors include the care receiver’s disability, cognitive impairment, and problem behaviours, as well as the high intensity of care, and some specific types of care tasks [10]. Moreover, caregivers’ burden increases as the care recipient’s stage of dementia progresses, especially when they become increasingly dependent, requiring full-time assistance with ADL (activities of daily living: personal hygiene, dressing, eating, maintaining continence, mobility) and IADL (instrumental activities of daily living: basic communication, transportation, meal preparation, shopping, housework, medication management, personal finance management). Requiring such complex assistance also results in the carer’s missed hours at work and less time for socialisation with friends and family [11]. Many societies are still not prepared to support dementia care recipients at home, which results in a heavy objective burden on caregivers and, in many cases, in the need for a paid caregiver [12]. Therefore, the caregiver burden has implications for both the caregiver and the care recipient, as a compromised ability to provide care affects both the care and the quality of life of the persons cared for, increasing the risk of institutionalisation [13]. Our comparative study focuses on the family carers of older people living with dementia as (1) Italy and Hungary belong to different care regimes. Indeed, Italy represents a family-based (Mediterranean) care regime, where informal care, even supported by paid migrant care workers, is the main pillar of care, while Hungary is characterised by an Eastern European care regime, with very limited resources allocated to formal long-term care provision and a high reliance on family care; (2) In Italy and Hungary, the severity of dementia differed during the first wave of the COVID-19 pandemic (see below).

### 1.1. The Effects of the COVID-19 Pandemic on Informal Carers

In terms of infections and deaths, Italy was one of the most affected, while Hungary was one of the least affected European countries during the first wave of the pandemic. According to WHO [14] data, the highest daily number of new cases and deaths per one million inhabitants in Italy was nine times greater than the corresponding Hungarian figures (smoothed new cases: IT: 93.16, HU: 10.44; smoothed new deaths: IT: 13.77, HU: 1.33).

European governments implemented a wide range of responses and measures to tackle the pandemic. The so-called Government Response Stringency Index (Hale et al., 2020) is aimed at indicating the strictness of the “lockdown-style” policies. The index, which has a value from 0 to 100 (100 = strictest response), is a composite measure of nine response indicators including school closures, workplace closures, and travel bans, recording the strictness of government policies. On the date the WHO declared COVID-19 a pandemic (11 March 2020), only Italy had a high stringency index score (85.9) in Europe, while Hungary had much lower scores (between 40 and 50, together with other Eastern European countries).

The Italian government adopted a more restrictive set of measures between January and July 2020, being the first European country to put a comprehensive lockdown in place.

The declared state of emergency was much longer in Italy, initially spanning six months from 31 January until 31 July. A state of emergency was declared in Hungary on 11 March 2020 and lasted until 18 June of the same year.

Several measures were similar in both countries in the first wave, and they correspond to different components of the stringency index. One substantial difference between the two countries was that in Hungary, shopping from 9 a.m. until 12 a.m. was allowed only for those over 65 years of age, while in Italy there was no similar restriction. There was no limitation on the use of transportation between municipalities in Hungary, while there was a strict lockdown between municipalities in Italy. Individuals and persons living in the same household were allowed to exercise outside in Hungary, while in Italy, sports activities were not allowed.

In short, stringency measures during the first wave of the pandemic covered a shorter period in Hungary, and in general citizens enjoyed more freedom compared to Italy.

The COVID-19 pandemic negatively affected the health, well-being, and wellness of people worldwide, and studies have highlighted that informal caregivers, representing a key component of care provision to older people with long-term care needs in contemporary societies, are a population group experiencing a significant increase in care burden, as well as social isolation and emotional stress [15]. The pandemic seems to expose the informal caregivers of people living with dementia to an especially high risk of negative health effects and worsening well-being, since physical problems and depression were already more prevalent among them before the COVID-19 outbreak compared to the informal caregivers of people without dementia [16,17,18]. This is confirmed by the results of a rapid systematic review [19], which highlighted that the COVID-19 pandemic has had a negative impact on both the health and well-being of the informal caregivers of older people with dementia during the health crisis.

Other studies showed that the informal carers of people with dementia reported a significant increase in anxiety, depression, distress, and irritability related to the policy restrictions put in place for tackling the COVID-19 outbreak [20], the pandemic quarantine [21], and the length of time in isolation [22]. Panerai et al. [23] pointed out that home confinement due to the COVID-19 outbreak increased the risk of burnout by 10% for the informal caregivers of older people with dementia. Cohen et al. [24] found that social isolation due to the pandemic confinement increased the stress of informal carers regardless of the dementia stage of care recipients, though carers providing assistance to severe cases experienced more stress. Due to the pandemic, mental health and psychological problems (e.g., anxiety and depression) have been particularly prevalent among the informal carers of people with dementia, who had to endure the additional burden of social isolation during the outbreak, an increasing problem for both family carers and the people they care for. Quarantines, lockdowns, “stay-at-home” policies, and the interruption or the cancellation of social, health, and community support services for older people with long-term chronic conditions have deepened the isolation of all parties concerned, and have intensified the related psychological effects. Thus, a new risk factor for the health of the informal carers of people with dementia (and the persons they care for) related to the COVID-19 outbreak is home confinement, resulting in social isolation [19].

The pandemic also had a negative impact on other aspects of the informal carers’ lives, such as the types and levels of worries experienced by the family carers of people with dementia and with other health problems. For example, a European survey carried out among about 2500 informal carers of older, frail, or disabled people (including care recipients with dementia) showed that 90.6% of respondents were worried about what would happen to the cared-for person should the carer have to self-isolate or become ill due to a COVID-19 infection. About four out of five carers (78.2%) were worried about a possible decline in the physical and mental health of their loved ones due to the COVID-19 outbreak. Moreover, about four out of ten carers (41.8%) worried about their ability to carry out care tasks safely due to a lack of COVID-19-related knowledge, information, or equipment [25]. Other studies found that informal carers were worried about the fact that services that supported them prior to COVID-19 may not become available again [26], about the financial impact of the lockdown [27], about the long-term impact of COVID-19-related restrictions on their relatives [28], and about what would happen to the care recipients should they become unable to care for them [27,28,29,30]. Moreover, other studies highlighted that government communication strategies (i.e., the unavailability of good and comprehensible information on how to deal with the pandemic might negatively affect carers’ ability to respond to the outbreak) and increased mass media and social media exposure during the pandemic could have worsened the worries, fears, and mental health vulnerabilities of informal caregivers (including those providing care to older people with dementia). This might have led to increasing mental health problems (e.g., anxiety, depression) among them [31,32].

### 1.2. Dementia Care in Italy and Hungary during the COVID-19 Pandemic

According to Tur-Sinai et al. [12], who identified different clusters of European countries based on their reactions to the COVID-19 crisis, both Italy and Hungary belonged to clusters of countries showing challenges concerning the degree of resilience, with Italy being part of a cluster having weak resilience in informal care and moderate resilience in formal care provision, and Hungary belonging to a cluster with weak resilience in both formal and informal care. Around 1.3 million people were estimated to have dementia in Italy in 2018, representing 2.1 per cent of the population, and 9% of those aged 65 or over [33]; about 80% are assisted directly by a family member. Overall, it is estimated that there are about 3 million people directly or indirectly involved in caregiving for people with dementia [34].

Between 146,000 [33] and 250,000 [35] people were estimated to have dementia in Hungary in 2018, representing 1.5 per cent of the population, and 7.4 per cent of those aged 65 or over [33]. The total number of family carers is estimated to be between 400,000 and 500,000 [35].

As shown in a previous paper [36] written by the authors of the present study on changes in the support system of Italian and Hungarian family carers of dementia patients during the first wave, the pandemic resulted in a substantial increase in the psychological and physical burden on carers in both countries, likely related to a drop in the utilisation (and availability) of care-related support services and aids. Despite the differences in the dementia care systems, the severity of the first wave of the pandemic, and the stringency measures adopted by the Italian and the Hungarian governments putting the two countries on different trajectories, the number of family carers left with no external help rose in both samples. The pandemic affected the two countries differently in terms of the deterioration of the state of the cared-for person, which was reported more frequently in the Italian sample (especially emotional regulation problems, such as aggression and apathy), and cognitive impairment (not recognising family members, confusion, and a worsening general mental state) and physical deterioration, which were more prevalent in the Hungarian sample. Italian carers also experienced more severe financial difficulties and a larger drop in the utilisation of external care-related help (in particular, paid carers and other family members).

These findings highlighted the systemic weaknesses of support structures for the family carers of people living with dementia, some of which are country-specific. Before the COVID-19 lockdown, Italian family carers relied heavily on help from paid (mostly migrant) care workers (and to a lesser extent either on daycare centres or on services provided by municipalities) in order to support their older loved ones living with dementia. As a result, the interruption of healthcare and social support services, as well as the border closures affecting the free movement of migrant care workers, made Italian carers extremely overwhelmed. Although the Hungarian system was much less supportive of the family carers of dementia patients from the outset, it was less vulnerable to border closures, due to mainly relying on local actors and the families themselves.

### 1.3. The Aim of the Present Study

The COVID-19 outbreak made it more difficult (and sometimes impossible) for people living with dementia and their caregivers to access the support systems and financial aid implemented by most of the countries to support them, partly due to the mandatory stringency measures adopted by governments during the first wave of the pandemic. Drawing upon the yet-unpublished results of the authors’ previous online survey research [36], the present paper compares two countries with different care regimes (Italy: family-based, Hungary: Eastern European), with the aim to answer the following research questions:

1. How did the pandemic change the subjective health of carers, and what were their experiences with care-related worries and workload?
2. What factors significantly predicted negative changes in these experiences?
3. What were carers’ main difficulties during the first pandemic wave?

## 2. Materials and Methods

### 2.1. Data Collection

The online questionnaire was designed for this research using Google Forms. It was distributed in both countries via social media channels (self-help Facebook groups and pages of family carers of people with dementia). Keywords used for finding the appropriate Facebook groups included Alzheimer’s, dementia, Parkinson’s, carers’ self-help groups, and daycare centres. The link to the questionnaire was shared in 33 Italian Facebook groups and pages totalling 43,566 members, and in 6 Hungarian Facebook groups totalling 11,344 members. Data were collected between May and July 2020. The objective was set at a minimum sample size of 150 respondents per country, in order to be able to carry out at least basic statistical calculations.

### 2.2. Questionnaire

The survey consisted of questions related to the socio-demographic background of both the family caregiver and the cared-for person; aspects of the carer–care receiver relationship (and pandemic-related changes in this relationship); carers’ responsibilities; carers’ self-reported physical, general, and mental health status, including the level of stress experienced due to the pandemic; the type and source of help the carer received with care-related activities, both before and after the declared state of emergency; types of physical and mental resources carers were able to utilise in order to cope with the difficulties posed by the pandemic; and helping factors and problems in carrying out care tasks. The questionnaire was presented in its original form (in Hungarian) to Hungarian respondents. For the Italian field study, it was translated into English, and after cross-checking, from English into Italian. The questionnaire was created by the authors for this research project specifically. No questionnaires of other authors were used.

### 2.3. Sample

Selection criteria in both countries included being the primary family carer of a non-institutionalised older person with dementia. Carers of dementia patients who were living in nursing homes, as well as carers who were not family members, were excluded from the sample.

The sample consisted of 188 Italian and 182 Hungarian respondents (88% female and 12% male) with an average age of 54 years. A total of 88% of respondents were female and 12% male, which is in line with the literature in terms of the long-term home care of older persons [37,38,39], and results concerning the informal carers of people living with dementia [40,41]. Nearly all respondents (90% of the Italian and 99% of the Hungarian respondents, respectively) were educated at least up to the secondary level, and most of them (64%) lived in cities—although this number was notably smaller in the Italian sample, where 49% reported living in rural areas or villages. Two-thirds of respondents (69%) reported being married or in a relationship, whereas 31% reported being single, widowed, or divorced. Most respondents were either the child (73%) or the partner (17%) of the cared-for person, and more than half of them (58%) lived together with the older person living with dementia before the pandemic (although this number was significantly higher among Hungarian respondents than among Italian ones, 65% per cent and 52% per cent, respectively). A total of 66% per cent of the family carers participating in the study reported being currently employed. Over half (54%) of the respondents reported that the person they cared for was 80 years of age or more, with another one third (32%) reporting an age of between 70 and 79 years. Around 15% per cent of family carers were caring for someone younger than 70 years of age. Most respondents reported caring for someone with either a medium (44%) or high (42%) severity of dementia, however, high severity was significantly more common in the Italian (52%) than in the Hungarian (32%) sample (χ^2^(1) = 14.785; *p* < 0.001; Phi = 0.200).

### 2.4. Statistical Data Analysis

Participant demographic characteristics and background variables were analysed using frequency analysis. Five-point Likert scales were used to measure general and mental health changes and subjective workload. A scale constructed from three statements measured on a 5-point Likert scale was used to indicate pandemic-related worry. Due to the non-normal distribution of these wellbeing variables, nonparametric tests were used to assess deviation from the middle of the scale (one-sample Wilcoxon’s signed-rank test), and cross-country comparison (Mann–Whitney’s U test). Open-ended questions coded into non-exclusive categories were used to survey the problems encountered by family carers during the pandemic. Chi-square tests and Spearman’s rho were used to calculate associations between the wellbeing variables and background factors measured in the questionnaire. The variables with significant associations were then included as predictors in binary logistic regression models (one for each of the four wellbeing scales). Binary logistic models were chosen because we were interested in only one end of these scales (the worsening in the case of general and mental health, and the high end in the case of subjective overload and worry), therefore the scales were recoded to dichotomous variables before being put into the regression models.

The software used for running statistical analyses was IBM SPSS Statistics (IBM Corp. Released 2019. IBM SPSS Statistics for Windows, Version 26.0.0.0, IBM Corp., Armonk, NY, USA), and the significance level was set at *p* < 0.05.

## 3. Results

### 3.1. Indicators of Family Carers’ Wellbeing

#### 3.1.1. General and Mental Health Changes

We used the following question to measure family carers’ change in general health during the first wave of the pandemic: “Did you experience a change in your health since the declaration of the emergency? (1—yes, my health worsened a lot, 5—yes, my health improved a lot)”. We asked carers to rate the change on a one-to-five-point Likert scale, where lower numbers meant a negative change and higher numbers a positive change. When interpreting the results, we considered values above 3 to be a change in the positive direction, values below 3 in the negative direction, and the value “3” itself as no change.

The mean change in general health was 2.54 (SD = 0.98) in the Italian sample and 2.97 (SD = 0.89) in the Hungarian sample. A one-sample Wilcoxon signed-rank test indicated that the median was only significantly lower than the middle of the scale in the Italian sample (Italy: Z = −5.9; *p* < 0.001, Hungary: Z = −0.574; *p* = 0.566), meaning that in the Italian sample, more carers experienced a deterioration instead of an improvement, and in the Hungarian sample, general health remained the same on average. Hungarian carers had significantly better general health outcomes than Italians (Mann–Whitney’s U = 13,095.0; Z = −4.163; *p* < 0.001; η^2^ = 0.05), and significantly more Italian (41%) than Hungarian (26%) carers reported a worsening in their general health (χ^2^ = 9.505; *p* = 0.002; Phi = 0.160).

#### 3.1.2. Mental Health Changes

We used the following question to measure family carers’ change in mental health during the first wave of the pandemic: “How did the difficulties posed by the pandemic affect your mental health? (1—it is worse than before the pandemic, 5—it is better than before the pandemic)”. We asked carers to rate the change on a one-to-five-point Likert scale, where lower numbers meant a negative change and higher numbers a positive change. When interpreting the results, we considered values above 3 to be a change in the positive direction, values below 3 in the negative direction, and the value “3” itself as no change.

The mean change in mental health was 2.25 (SD = 0.93) in the Italian sample and 2.55 (SD = 0.99) in the Hungarian sample. A one-sample Wilcoxon signed-rank test indicated that the median was significantly lower than the middle of the scale (Italy: Z = −8.203; *p* < 0.001, Hungary: Z = −5.570; *p* < 0.001), meaning that in both samples, more carers experienced deterioration than improvement. Hungarian carers had significantly better general health outcomes than Italians (Mann–Whitney’s U = 14,041.0; Z = −3.168; *p* = 0.002; η^2^ = 0.03), and significantly more Italian (55%) than Hungarian (39%) carers experienced a decline in their mental health (χ^2^ = 9.867; *p* = 0.002; Phi = 0.163).

#### 3.1.3. Pandemic-Related Worry

In order to create a simple yet illustrative scale describing the worries experienced by family carers during the pandemic, we used three statements covering the two most problematic areas for carers: worry for the health of the cared-for person, and the unavailability of services utilised before the pandemic. The statements were as follows: “During the past week...”

1. “I worried about the health of the person I care for”;
2. “I worried about infecting the person I care for”;
3. “The narrowing of the access to services gave me anxiety”.

We asked respondents to rate the frequency of the above three experiences on a scale ranging from 1 to 5, where the value “1” corresponded to “almost never” and “5” to “always”.

The worry scale was created by taking the mean of the three numbers belonging to the three questions, keeping the value structure of the original statements. The scale’s inner consistency was acceptable with a Cronbach’s alpha of 0.638.

The mean worry-value was 4.2 (SD = 0.93) in the Italian sample and 3.7 (SD = 0.89) in the Hungarian sample. A one-sample Wilcoxon signed-rank test indicated that the median was significantly higher than the middle of the scale (Italy: Z = 10.473; *p* < 0.001, Hungary: Z = 8.185; *p* < 0.001), meaning that in both samples, carers experienced high worry levels. Significantly more Italian (74%) than Hungarian (43%) carers reported a high worry-level (a value of 4.0 or above) (χ^2^(1) = 36.831, *p* < 0.001, Phi = −0.316).

#### 3.1.4. Subjective Workload (Feeling Time-Constrained)

In order to measure how overwhelmed carers felt during the pandemic, we asked them to rate how often they felt time-constrained between their tasks during the past week. The statement was worded as follows: “During the past week I felt time-constrained among my many tasks.” Possible responses ranged from 1 to 5, where the value “1” corresponded to “almost never” and “5” to “always”.

The mean subjective workload value was 3.2 (SD = 1.3) in the Italian sample and 3.7 (SD = 1.3) in the Hungarian sample. A one-sample Wilcoxon signed-rank test indicated that the median was significantly different from 3.0 (the middle of the scale) (Italy: Z = 2.098; *p* = 0.036, Hungary: Z = 6.156; *p* < 0.001), meaning that in both samples, more carers felt overwhelmed than not. The subjective workload was significantly higher in the Hungarian sample (Mann–Whitney’s U = 13,587.0; Z = −3.531; *p* < 0.001; η^2^ = 0.034). Significantly more Hungarian (56%) than Italian (40%) carers reported feeling highly overwhelmed (a value of 4.0 or above) (χ^2^(1) = 9.666, *p* = 0.002, Phi = 0.162).

#### 3.1.5. Problems Mentioned in an Open-Ended Question

We used the following open-ended question to get a grasp of what family carers themselves considered to be their biggest problems related to the pandemic: “*Please list a maximum of five things that constitute a problem for you during the pandemic*”. We then coded the raw responses and grouped these codes into meaningful categories. Table 1 displays the codes and the categories (asterisks indicate a significant difference between the two samples).

Codes are not exclusive. Carers listed an average of 3.05 problems (SD = 1.86) in the Italian sample and an average of 2.25 problems (SD = 1.8) in the Hungarian sample. Italian carers mentioned significantly more problems (Mann–Whitney’s U = 12,642.00, Z = −4.409, *p* < 0.001).

### 3.2. General Health Deterioration of Carer

#### 3.2.1. Factors Related to Carer’s General Health Deterioration

The general health deterioration of carers was linked to the following factors in both samples:

- The deterioration of the state of the person with dementia;
- Carers’ mental health deterioration;
- The decline in the carer–care receiver relationship;
- The carer’s poor pre-pandemic health;
- The high number of the care receiver’s deterioration symptoms;
- The high level of the carer’s pandemic-related worry.

Only in the Italian sample:

- Abandonment is mentioned among the problems;
- The carer not receiving any help during the first wave of the pandemic despite needing it;
- An increase in the carer’s care time;
- A deterioration in the emotional regulation capabilities of the care receiver;
- The carer’s physical or mental deterioration is mentioned among the problems;
- Shopping is among the carer’s tasks;
- Managing official affairs on behalf of the care receiver is among the carer’s tasks;
- Not having help from family during the first wave of the pandemic;
- Not gaining new care-related help from family (if the carer did not have this type of help before the pandemic);
- Having lost the help the carer had for everyday tasks (any one of: housework, personal hygiene of the dementia patient, daytime surveillance) before the pandemic;
- Feeling highly time-constrained between tasks (high subjective overwhelmedness).

Only in the Hungarian sample:

- The physical deterioration of the care receiver;
- Being the partner or other relative of the care receiver (instead of their child);
- Moving the patient is among the carer’s tasks;
- Feeding is among the carer’s tasks;
- Cooking is among the carer’s tasks;
- Bathing the care receiver is among the carer’s tasks;
- Needing external care-related help during the first wave of the pandemic;
- The older age of the carer;
- A high number of care tasks.

Detailed statistical calculations for the factors listed above can be found in Table A1 of Appendix A.

#### 3.2.2. Logistic Regression Model

We used the factors significantly linked to a deterioration in the carer’s general health in a binary logistic regression model (conditional forward stepwise method) to find out which of these factors had partial predictive power. The final models are presented in Table 2.

The following variables entered the model in the Italian sample (χ^2^(6) = 84.316, *p* < 0.001, Cox and Snell R Square = 0.443, Nagelkerke R Square = 0.593):

- Carer’s mental health deterioration (linked to a higher likelihood of deterioration);
- Carer’s pre-pandemic health (bad health linked to a higher likelihood of deterioration);
- Shopping is among the carer’s tasks (linked to a higher likelihood of deterioration);
- Gaining new care-related help from family (if the carer did not have this type of help before the pandemic) (linked to a lower likelihood of deterioration);
- Managing official affairs on behalf of the care receiver is among the carer’s tasks (linked to a higher likelihood of deterioration);
- Decline in the carer–care receiver relationship (linked to a higher likelihood of deterioration).

The model correctly predicted 80% of cases where a deterioration in general health was present and 85% of cases where no deterioration in general health was present, giving an overall correct prediction rate of 83%.

The following variables entered the model in the Hungarian sample (χ^2^(3) = 71.612, *p* < 0.001, Cox and Snell R Square = 0.390, Nagelkerke R Square = 0.578):

- Carer’s mental health deterioration (linked to a higher likelihood of deterioration);
- Carer’s pre-pandemic health (bad health linked to a higher likelihood of deterioration);
- Cooking is among the carer’s tasks (linked to a higher likelihood of deterioration).

The model correctly predicted 58% of cases where a deterioration in general health was present and 97% of cases where no deterioration in general health was present, giving an overall correct prediction rate of 88%.

### 3.3. Mental Health Deterioration of Carers

#### 3.3.1. Factors Linked to the Mental Health Deterioration of Carers

The mental health deterioration of carers was linked to the following factors in both samples:

- Carer’s general health deterioration;
- Decline in the carer–care receiver relationship;
- The deterioration of the state of the person with dementia;
- An increase in the carer’s care time;
- The carer’s physical or mental deterioration is mentioned among the problems;
- The carer’s poor pre-pandemic health;
- The high number of the care receiver’s deterioration symptoms;
- The high level of the carer’s pandemic-related worry.

Only in the Italian sample:

- Abandonment is mentioned among the problems;
- A deterioration in the emotional regulation capabilities of the care receiver;
- Carer not receiving any help during the first wave of the pandemic despite needing it;
- Needing external care-related help during the first wave of the pandemic;
- Losing all care-related help that the carer used to receive before the pandemic.

Only in the Hungarian sample:

- The physical deterioration of the care receiver;
- Not having had any care-related help from healthcare providers (any one of: family doctor, specialist, medical assistant, ambulance) during the first wave of the pandemic;
- Lack of care-related help from the family doctor during the first wave of the pandemic;
- Receiving volunteer care-related help (from any one of: charities, church, colleagues, neighbours, friends, volunteers, telephone helpline) during the first wave of the pandemic;
- Having left full-time employment during the first wave of the pandemic;
- Moving the patient is among the carer’s tasks;
- Financial difficulties during the first wave of the pandemic;
- Having feeding among the carer’s tasks;
- A high number of care tasks.

Detailed statistical calculations for the factors listed above can be found in Table A2 of Appendix A.

#### 3.3.2. Logistic Regression Model

Like with general health, we used the factors significantly linked to the deterioration in the carer’s mental health in a binary logistic regression model (conditional forward stepwise method) to find out which of these variables have partial predictive power. The final model is presented in Table 3.

The following variables entered the model in the Italian sample (χ^2^(6) = 71.234, *p* < 0.001, Cox and Snell R Square = 0.388, Nagelkerke R Square = 0.524):

- Carer’s general health deterioration (linked to a higher likelihood of mental health deterioration);
- Pandemic-related worry levels of carer (higher worry linked to a higher likelihood of mental health deterioration);
- A deterioration in the emotional regulation capabilities of the care receiver (linked to a higher likelihood of mental health deterioration);
- Abandonment is mentioned among the problems (linked to a higher likelihood of mental health deterioration).

The model correctly predicted 83% of cases where there was a deterioration in mental health and 73% of cases where there was not, giving an overall correct prediction rate of 79%.

The following variables entered the model in the Hungarian sample (χ^2^(4) = 69.273, *p* < 0.001, Cox and Snell R Square = 0.390, Nagelkerke R Square = 0.529):

- Carer’s general health deterioration (linked to a higher likelihood of mental health deterioration);
- Pandemic-related worry levels of carer (higher worry linked to a higher likelihood of mental health deterioration);
- A decline in the carer–care receiver relationship (linked to a higher likelihood of mental health deterioration);
- Receiving care-related help from the family doctor during the first wave of the pandemic (linked to a higher likelihood of mental health deterioration).

The model correctly predicted 69% of cases where there was a deterioration in mental health, and 91% of cases where there was not, giving an overall correct prediction rate of 82%.

### 3.4. Pandemic-Related Worry

#### 3.4.1. Factors Linked to the High Pandemic-Related Worry Levels of Carers

The high pandemic-related worry levels of carers were linked to the following factors in both samples:

- Being the child of the care receiver;
- Feeling highly time-constrained between tasks (high subjective overwhelmedness).

Only in the Italian sample:

- Not being the partner or other relative of the care receiver (instead, being their child or other relative);
- Having had and then having lost the help received with daytime surveillance (instead of never having it or not losing it);
- Carer’s general health deterioration;
- Anxiety is mentioned among the problems;
- Carer not being retired;
- Having stayed in full-time employment during the first wave of the pandemic;
- Having changed to working from home during the first wave of the pandemic;
- Carer not receiving any help during the first wave of the pandemic despite needing it;
- Younger age of the carer.

Only in the Hungarian sample:

- The deterioration of the state of the person with dementia;
- The physical deterioration of the care receiver;
- An increase in the carer’s care time;
- A decline in the carer–care receiver relationship;
- Time management is mentioned among the problems;
- Conversation, communication is among the carer’s tasks;
- The patient’s worsening quality of life is mentioned among the problems;
- A higher number of the care receiver’s deterioration symptoms.

Detailed statistical calculations for the factors listed above can be found in Table A3 of Appendix A.

#### 3.4.2. Logistic Regression Model

We used the factors significantly linked to a high level of pandemic-related worry in a binary logistic regression model (conditional forward stepwise method) to find out which of these variables have partial predictive power. The results are presented in Table 4.

The following variables entered the model in the Italian sample (χ^2^(4) = 37.903, *p* < 0.001, Cox and Snell R Square = 0.213, Nagelkerke R Square = 0.317):

- Being the child of care receiver (linked to a higher likelihood of high worry levels);
- Carer’s general health deterioration (linked to a higher likelihood of high worry levels);
- Having lost the help the carer had for daytime surveillance before the pandemic (linked to a lower likelihood of high worry levels);
- Anxiety is mentioned among the problems (linked to a higher likelihood of high worry levels).

The model correctly predicted 88% of cases of high worry and 49% of cases of medium or low worry, giving an overall correct prediction rate of 78%.

The following variables entered the model in the Hungarian sample (χ^2^(4) = 45.693, *p* < 0.001, Cox and Snell R Square = 0.275, Nagelkerke R Square = 0.373):

- The physical deterioration of the care receiver (linked to a higher likelihood of high worry levels);
- Feeling highly time-constrained between tasks (linked to a higher likelihood of high worry levels);
- Increase in the carer’s care time (linked to a higher likelihood of high worry levels);
- Having conversation, communication among the carer’s tasks (linked to a higher likelihood of high worry levels).

The model correctly predicted 61% of cases of high worry and 84% of cases of medium or low worry, giving an overall correct prediction rate of 75%.

### 3.5. Feeling Time Constrained among Tasks (Subjective Overwhelmedness)

#### 3.5.1. Factors Linked to Carers Feeling Highly Overwhelmed

The high subjective overwhelmedness of carers was linked to the following factors in both samples:

- High worry levels.

Only in the Italian sample:

- Having newly moved in together with the care receiver during the first wave;
- The decline in the carer–care receiver relationship;
- Carer’s general health deterioration;
- An increase in the carer’s care time;
- Carer not receiving any help during the first wave of the pandemic despite needing it;
- A deterioration in the emotional regulation capabilities of the care receiver;
- Losing all care-related help that the carer used to receive before the pandemic;
- Having had care-related help with mental health (any one of: conversation, emotional support) during the first wave of the pandemic;
- The carer’s physical or mental deterioration is mentioned among the problems;
- Being an inexperienced carer (caring for care receiver for 1 year or less);
- Financial difficulties during the first wave of the pandemic;
- Not having had care-related help from the family doctor during the first wave of the pandemic.

Only in the Hungarian sample:

- Time management is mentioned among the problems;
- Being the child of the care receiver;
- Having jobs around the house among the carer’s tasks;
- Having lost the help received from social service providers before the pandemic;
- Not having had any care-related help from social service providers during the first wave of the pandemic;
- Having cleaning among the carer’s tasks.

Detailed statistical calculations for the factors listed above can be found in Table A4 of Appendix A.

#### 3.5.2. Logistic Regression Model

We used the factors significantly linked to feeling highly overwhelmed in a binary logistic regression model (conditional forward stepwise method) to find out which of these variables have partial predictive power. The results are presented in Table 5.

The following variables entered the model in the Italian sample (χ^2^(4) = 31.221, *p* < 0.001, Cox and Snell R Square = 0.194, Nagelkerke R Square = 0.261):

- Pandemic-related worry levels (higher worry linked to a higher likelihood of feeling very time-constrained between tasks);
- Decline in the carer–care receiver relationship (linked to a higher likelihood of feeling very time-constrained between tasks);
- Having had some kind of care-related help at all during the first wave of the pandemic (linked to a lower likelihood of feeling very time-constrained between tasks);
- Having moved in together with the care receiver during the first wave of the pandemic (linked to a higher likelihood of feeling very time-constrained between tasks).

The model correctly predicted 53% of cases of high levels of time constraint and 77% of cases of medium or low levels of time constraint, giving an overall correct prediction rate of 67%.

The following variables entered the model in the Hungarian sample (χ^2^(4) = 37.651, *p* < 0.001, Cox and Snell R Square = 0.192, Nagelkerke R Square = 0.2571):

- Pandemic-related worry levels (higher worry linked to a higher likelihood of feeling very time-constrained between tasks);
- The carer had care-related help from social service providers during the first wave of the pandemic (linked to a lower likelihood of feeling very time-constrained between tasks);
- Being the child of the care receiver (linked to a higher likelihood of feeling very time-constrained between tasks);
- Jobs around the house are among care tasks (linked to a higher likelihood of feeling very time-constrained between tasks).

The model correctly predicted 77% of cases of high levels of time constraint and 56% of cases of medium or low levels of time constraint, giving an overall correct prediction rate of 68%.

## 4. Discussion

In this study, we examined the impact of the COVID-19 pandemic and the related restrictions on the subjective physical and mental health, worries, and problems of the family carers of people with dementia in Italy and Hungary.

### 4.1. Physical and Mental Health

As the results showed in both samples, both the health status prior to the pandemic and the change in the state of care receivers were significantly associated with carers’ health deterioration during the pandemic. The deterioration in the state of the persons living with dementia was likely related to the course of the pandemic and the severity of the restrictions introduced in response. It also seems probable that the lockdowns, the closure of daycare centres for persons with dementia, and the absence of paid carers had a negative impact on the state of persons living with dementia in both countries. According to the literature, the lack of social interactions, the disruption of daily routines, and the restriction of opportunities for physical activity (e.g., not being able to leave home or take a walk on the street or in nature) contribute to the more rapid deterioration of the state of dementia patients [42,43,44]. Makra and Balogh [45] pointed out that the findings of international research have shown an unequivocal connection between physical activity and cognitive functions. Moreover, reduced physical activity and social contact experienced during confinement hastened (with long-term impact) cognitive deterioration and the worsening of neuropsychiatric symptoms in dementia patients [46]. In the Italian sample, the percentage of persons reporting a deterioration in the state of care receivers was almost twice as high as in the Hungarian sample (62% compared to 37%, respectively), which could have been due in part to the fact that stricter conditions were imposed in Italy with a complete ban on leaving the home, while in Hungary the restrictions were less severe (people could go out to the street, go for long walks, exercise alone or with family members outdoors). Indeed, as noted in a previous study [36], and according to the Government Response Stringency Index [47], after the COVID-19 outbreak, the containment measures put in place in Italy by the government as mentioned were stricter, wider and longer than those in Hungary and across Europe (data collection was carried out during the first wave). This has disrupted the provision of care services. Italian older people with dementia and their carers were left without adequate support, which negatively affected their health and well-being.

The deterioration in the state of persons living with dementia likely increased the volume of nursing tasks, imposing additional physical and mental strain on carers, in times when outside help was less available (43% of the Italian and 29% of the Hungarian carers reported not having any care-related assistance during the first pandemic wave, an increase compared to pre-pandemic values of and 18% and 20%, respectively). Based on the results of a regression analysis, the general health deterioration of the carer can partly be predicted by the carer having certain tasks (shopping and managing the care receiver’s official affairs in Italy and cooking in Hungary), and the lack of deterioration can be predicted by having extra help from family—these findings also point to the role of workload in the carer’s health deterioration. The growing burden of care was probably the most impacting factor deriving from the lockdown. Studies have highlighted that intensive care and the unmet demands of psychological help and support in daily living activities are factors often correlated with higher perceived burden [48]. This is confirmed in our study: an increase in care hours correlates with worsening mental health. Compared to Italian carers, a smaller proportion of Hungarian carers reported a deterioration in their own health, which could be explained in part by the fact that the milder restrictions did not obstruct access to external help as much as in Italy. In the Italian sample, the increase in the proportion of carers who received no help was three times greater than in the Hungarian sample. One of the reasons for this could be that the family, as the most important support structure, was still able to help in Hungary (54% of Hungarian carers still received help from family during the first wave, whereas only 37% of Italian respondents reported the same) [48].

As the findings show, far more Italian (55%) than Hungarian (38%) carers experienced a negative change in their subjective mental state. One of the explanations for this could be the reduction or absence of social support during the pandemic. This is confirmed by the fact that, in a previous study based on the same sample [36], the Italian sample of carers experienced a significant and higher decrease in the utilisation of social services (i.e., social service providers, council, day care centres), compared to the Hungarian carers. The interruption of health and social services that made care work increasingly difficult may have been associated with mental distress [49,50]. Moreover, the inability to receive other forms of support, due to mobility restrictions, may have further increased the burden [51]. The findings indicate that in Italy, the stronger restrictions (a complete ban on leaving the home) significantly influenced/reduced the possibility of care support provided by others. Other researchers also confirmed the connection between the low level of social support and the increase in care burden, and the resulting deterioration in mental health [20].

Our study also found that the state of the care receivers living with dementia also determined the change in the mental health of their carers. As we have shown, the Italian respondents in our sample reported having care receivers with more severe cases of dementia, and significantly more Italian family carers indicated that they noticed a deterioration in the state of their family member since the outbreak of the pandemic. This is in line with the results of a study [24] conducted in Argentina, where it was found that the burden of carers was higher after four weeks of lockdown, especially in cases where care receivers were in an advanced stage of dementia. Another study showed that Italian informal carers of people with dementia reported a significant increase in anxiety, depression, distress, irritability and caregiver burden from the pandemic quarantine, the latter being also associated with an acute worsening of clinical symptoms in patients with dementia [21]. This is in line with our results that the mental health deterioration of the carer could partly be predicted by various aspects of the family ties between carer and care receiver, such as problems with the care receiver’s emotion regulation capabilities, resulting in aggression or apathy (Italian sample), or a decline in the carer–care receiver relationship (Hungarian sample).

In a study carried out by Alexopoulos et al. [52], the distress felt by family carers during lockdown significantly influenced the memory problems and neuropsychiatric symptoms of care receivers living with dementia, which was similar to our findings with regard to Italian family carers. In contrast, in the Hungarian sample, the main factors were deterioration in the physical state of care receivers living with dementia and the high number of care tasks. This might be connected to the low level of physical activity on the part of care receivers that could be deduced from Hungarian responses to open-ended questions.

Altieri and Santangelo [20] found more symptoms of depression among the family carers of persons living with dementia, probably attributable to the decline in social support. According to our findings, the psychological well-being of family carers was undermined by the deteriorating state of care receivers, the absence of outside help, and the limited access to or absence of health and social services. These results imply a need to quickly reduce the burden that weighs on families, both acting to improve the health status of carers and providing relief for distress linked to the cognitive deterioration of the care that studies showed to be particularly burdensome [53,54].

### 4.2. Carers’ Worry Levels

According to the literature [55], worry is linked to the assumed future occurrence of something bad; that is, we worry that something that we would not like will happen (e.g., the care receiver falling ill). In general, the pandemic has had a negative effect on the level of worry of the family carers of older people. The results of a Finnish study of the family carers of older people [56] and a Slovenian online survey of informal carers [57] confirmed this. In both countries, one-third of family carers worried about the situation caused by the pandemic (FI: 34%, SL: 35%). Our results show a similar proportion of carers with high worry levels in the Hungarian sample (43%) and an even higher number in the Italian sample (74%), however, the differences might be contributed to the differences in the scale used for measuring worry levels. Our findings showed that in both countries, the mental state of the family carers of persons living with dementia was influenced by worries directly related to the COVID-19 crisis. A Greek research group examining the carers of patients with mild or severe neurocognitive disorders reached the same finding [52].

One explanation for the significantly higher worry levels in the Italian sample could be that the pandemic situation, as mentioned, was far more serious in Italy during the first wave. The epidemic data could explain why the Italian carers were more worried that they themselves or the persons they cared for would become infected. Another aspect is the greater media exposure to the pandemic crisis. Indeed, it has been highlighted that since the start of the COVID-19 outbreak, there has been a considerable increase in the use of mass media (e.g., TV, newspapers, radio, internet, etc.) and social media (e.g., Facebook, Twitter etc.) amongst the population in Italy (as well as at the international level). An association was found between frequent media and social media exposure during the pandemic and a high prevalence of mental health problems (e.g., anxiety, depression) or sleep disorders among the informal carers of people with dementia [58]. In line with this, a study carried out in Italy showed an association between the COVID-19 media exposure and anxiety (and subjective loneliness), suggesting that continuing to stay informed may amplify anxiety among people, carers included. We found a significant association in the Italian sample between the level of distress and the carer’s age and their relationship to the care receiver, meaning younger people caring for their parents worried more. This might be related to the fact that younger carers are still active in the labour market: over a quarter (26%) of carers physically went to work at least part-time during the first wave, and probably came into contact with more people, which might have been a reason for worrying more about infecting their care receivers (compared to carers who are older or retired). During the first wave, the absolute numbers and the proportion of registered COVID cases were much lower in Hungary than in Italy, therefore caregivers active in the labour market were likely not as worried about getting infected themselves.

The more stringent measures adopted in Italy, which were also of a longer duration than those in Hungary, are another factor that could have contributed to the greater distress felt by Italian carers. Because of the lockdown during the first wave of the pandemic, the Italian carers lost their external help (e.g., the support of family, paid carers and other health and social services), giving rise to further distress. This is confirmed by the significant association between a high level of distress and the fact that the carer received no help from anyone even though it would have been needed. The connection between a lack of support for the home care of persons living with dementia and the distress of carers was also found in a qualitative study [59] conducted in the UK among people living with dementia (*n* = 8) and the unpaid carers (including family carers) of persons living with dementia (*n* = 42). The study found that carers were worried about whether social support service provisions would re-open in the future, and whether the person they care for would still be able to access previously enjoyed services after they re-open.

In the Hungarian sample, we found that carers’ higher level of worry was related to a deterioration in the state (especially the physical state and health status) of the care receiver during the first wave. The reason for this could be that in April 2020, 60% of hospital beds were provisioned by the Hungarian Minister of Human Resources to be emptied in anticipation of the rise in the number of serious cases of COVID-19 [60], a measure criticised for not being justified by the pandemic figures, and for having serious adverse effects in terms of the unmet healthcare needs of non-COVID patients [61,62,63]. As a result, many—mainly older—patients in need of expert rehabilitation or supervision, some even requiring 24-hour nursing, had to leave the hospital. In addition, the number of doctor–patient encounters was restricted [64], non-life-saving operations were postponed [65], and it became impossible to access outpatient and other psychiatric, rehabilitation, and physiotherapy services (e.g., remedial exercises to improve the general condition) [66]. A number of carers expressed, in response to the open questions, that this placed their family members living with dementia and themselves in a difficult position. The higher worry levels of Hungarian family carers were significantly related to being responsible for specific tasks (e.g., shopping) and the increased care time. This could be contributed to shopping restrictions imposed by the government [67], restricting shopping to times between 9 and 12:00 a.m. for persons over 65, and imposing shorter opening hours in the evenings. As a result, younger carers faced waiting in queues to shop for food or collect prescribed medicines, and those who were working might have had to leave work earlier than usual to be able to get to the stores before they closed. Constant long waits and the crowding in the stores and pharmacies during after-work hours made social distancing more difficult, which could have contributed to the high worry levels of carers in the Hungarian sample.

### 4.3. Carers’ Subjective Overwhelmedness (Feeling Time-Constrained between Tasks)

Shopping, a relatively simple task carried out by most carers in the Hungarian sample, also became very time consuming due to restrictions, disturbing carers’ daily routines, and likely affecting other care tasks and care time in general. This might have contributed to the fact that subjective overwhelmedness (feeling time-constrained among tasks) was significantly higher in the Hungarian sample. The results of a Hungarian online survey study on the family carers of older people (*n* = 231) during the first wave of the COVID-19 pandemic confirmed these findings, as one-third of respondents indicated that the increase in care burden was related to government restrictions [68].

Our results show that carers’ subjective overwhelmedness is significantly predicted by carers’ worry levels in both samples. The “shrinking world” theory of Talbot and Briggs [69], describing the experience of dementia patients of their world shrinking after receiving the diagnosis, comes to mind. Talbot and Briggs theorised that the COVID-19 pandemic had accelerated this “shrinking world” effect on dementia patients—and our results indicate that this might be the case for carers as well. Closures and restrictions made carers feel that their usual places, activities and methods to recuperate became unavailable and that they were isolated and abandoned with no outside help. The shrinking-world theory seems to apply in both samples, manifesting in different ways. In the Italian sample, the deterioration of the carer–care receiver relationship, the complete lack of external help, and the carer moving in together with the care receiver were the factors predicting feeling time-constrained, all three fitting in well with the feeling of a shrinking world. In the Hungarian sample, the factors contributing to feeling time-constrained were the lack of help from social service providers (the main type of support apart from family), being the child of the care receiver, and having to do jobs around the house, all of them implying having to spend more time in the home of the care receiver, significantly narrowing the carer’s opportunities for doing other things.

## 5. Conclusions

The study examined the general and mental health, worry levels, and subjective overwhelmedness of the family carers of older people living with dementia during the first wave of the COVID-19 pandemic, in two countries with different—family-based (Italy) and Eastern European (Hungary)—care regimes.

In the present study, four different measures were applied to assess the circumstances of the family carers of people with dementia: general health, mental health, worry, and carers’ subjective overwhelmedness. Our data have shown that the deterioration of family carers’ physical or mental health potentially leads to the erosion of their personal resources utilised in care work. We underline also that the legislation might have played an important role. As mentioned above, a deterioration in the state of the care receiver leads to high levels of worry in both Italian and Hungarian carers, which are probably related to the legislation/norms put in place in both countries. For example, in Hungary, the legislation ordered hospitals to leave 60% of hospital beds at the disposal of pandemic patients. In Italy, the strict measures adopted for tackling the spread of the pandemic caused the interruption, postponement, and cancellation of social, health, and community services (e.g., daycare services) for older people with dementia and other LTC needs, making carers worried about where their loved ones might be placed should they require hospitalisation, or which service to rely on should they need health, social, or community care. Our results show that carers’ subjective overwhelmedness and worry levels go hand in hand in both samples.

Our conclusion is that the reduction in these resources may be compensated via alternative care solutions in the healthcare system and social services, e.g., by the restructuring of formal care, the incorporation of new elements and technical solutions, and their coordination in order to ease family carers’ burden. For example, for reducing subjective carers’ burden and supporting their resilience, it is necessary to develop comprehensive psychological interventions, while for tackling the objective burden, the supply of a more capillary respite service network should be implemented.

Moreover, more accessible information on available support services, such as counselling and ICT-based help solutions (whose role has increased during the pandemic), by means of a wide range of channels, e.g., general practitioners, should be guaranteed.

Our data was collected during the first wave of the COVID-19 pandemic, and no follow-up research has yet been conducted. Substantive practical and policy recommendations should be formulated based on further research with more recent data. However, it is clear from our study that decision-makers need to make it a priority to help vulnerable groups such as family carers, by reflecting on their needs and developing an action plan in case of emergencies.

We believe our study contributes to the scientific evidence base emerging on the subject worldwide. However, further comparative research is needed to understand how a country’s care regime alters the mechanism of action of the factors affecting the lives of family carers during the pandemic.

### Limitations of the Present Study

One of the limitations of this study is related to the data collection. An online questionnaire was distributed in self-help Facebook groups and pages, as family carers of older people with dementia are a hard-to-reach group, especially in the midst of the first wave of the COVID-19 pandemic. Online questionnaires have drawbacks as they prevent internet non-users from even seeing the call to participate in the study. Recruiting participants via Facebook groups and pages has its own pitfalls as well: it makes the success rate of reaching group members subject to an externally controlled website’s algorithm. Therefore, the sample of our study cannot—and does not intend to—be representative of the Hungarian or Italian family carers of people with dementia. The results cannot be considered representative in terms of socio-demographic characteristics, either. Another limitation is the relatively small sample size for a quantitative study, which makes viewing the conclusions in the context of other well-established findings necessary. A third limitation is using ad-hoc questions instead of well-reviewed scales for measuring physical and mental health and worry in order to limit the length of the questionnaire and consequently increase the number of completers. Finally, a fourth limitation is the retrospective analysis of change in carers’ health and mental health based on cross-sectional data, instead of comparing data points measured at different times in a longitudinal design. Despite these limitations, this study offers new insights for further understanding the challenges faced by the family carers of people with dementia.

## Figures and Tables

**Table 1:** Responses given to an open-ended question regarding problems encountered by carers during the pandemic and the frequency of mentions in the two subsamples.

Category	Codes Belonging to the Category	Italian Sample	Hungarian Sample
Medical and social care	- Medical care unavailable - Social care unavailable, social institutions closed	36%	38%
Shopping and medicine acquisition	- Shopping difficulties - Difficulty buying medicine, medical equipment and protective gear - Annoyance with infection prevention measures (e.g., mask, disinfection, gloves) - Queues, long waits in lines	21%	35% *
Restricted freedom	- Confinement, lack of free movement - Curfew, lockdown, and other restrictions	28%	20%
Isolation	- Isolation from friends, relatives, and communities, no personal contact, loneliness	30% *	18%
Anxiety	- Anxiety, fear, worry - Fear of infection (self or patient)	22% *	8%
Abandonment	- Abandonment, no help to care for patient, helplessness - Carer’s isolation from patient - Difficulty with care tasks	24% *	6%
Carer’s mental and physical deterioration	- Carer’s mental exhaustion, insomnia - Carer’s own health - Frustration, stress - Exhaustion, fatigue - Hopelessness, depression	14%	10%
Patient’s quality of life	- Difficulty keeping patient occupied, no social life for patient - Difficulty getting patient to understand the situation and abide by the rules (stay home, isolate, wear protective gear) - Dealing with patient’s emotions - No exercise for patient - Patient’s health and mental health deterioration	18% *	7%
Everyday commitments	- Financial problems, excessive expenses - Difficulty with admin tasks, post office and bank - Commute and travel difficulties - Unavailable services (e.g., hairdressers, repairs)	11%	14%
Carer–patient relationship	- Needing 24-hour supervision - Unbearable responsibility (for medical decisions the carer is not competent in) - Spending too much time with the patient - Family conflict, quarrels, decline in carer–patient relationship - Managing dementia symptoms	14% *	7%
Relaxation	- Inability to relax, lack of recreation and time for self - Lack of exercise for carer - Inability to attend religious services	14%	10%
Time management	- Work problems, work–care conflict, no work or work loss - Disrupted routines and everyday life, difficult time management - Clash with childcare and other family commitments - Clash with chores and housework commitments	14%	10%
Chaos	- No information, misinformation, uncertainty - Finding the state/government incompetent, debilitating effects of restriction measures - Other people not abiding by the rules	10%	5%
No problems encountered		4%	9%
Missing/invalid response		2%	4%

* Significant difference between the two samples.

**Table 2:** Variables with significant partial predictive power over carers’ general health deterioration (conditional forward stepwise method, final model).

	B	S.E.	Wald	df	Sig.	Exp(B)
*Italian sample*						
Carer’s mental health deterioration (yes/no)	−2.683	0.542	24.523	1	0.000	0.068
Carer’s pre-pandemic health (scale of 1 to 5)	1.258	0.334	14.172	1	0.000	3.520
Shopping is among the carer’s tasks (yes/no)	1.634	0.796	4.215	1	0.040	5.123
Gaining new care-related help from family (if the carer did not have this type of help before the pandemic) (yes/no)	3.043	1.411	4.651	1	0.031	20.966
Managing official affairs on behalf of the care receiver is among the carer’s tasks (yes/no)	1.450	0.678	4.569	1	0.033	4.262
Decline in the carer–care receiver relationship (yes/no)	−0.986	0.493	3.997	1	0.046	0.373
Constant	−2.458	1.169	4.417	1	0.036	0.086
*Hungarian sample*						
Cooking is among the carer’s tasks (yes/no)	3.146	1.063	8.768	1	0.003	23.252
Carer’s mental health deterioration (yes/no)	−2.956	0.594	24.742	1	0.000	0.052
Carer’s pre-pandemic health (scale of 1 to 5)	1.618	0.432	14.045	1	0.000	5.044
Constant	−3.286	1.430	5.279	1	0.022	0.037

**Table 3:** Variables with significant partial predictive power over carers’ mental health deterioration (conditional forward stepwise method, final model).

	B	S.E.	Wald	df	Sig.	Exp(B)
*Italian sample*						
Carer’s general health deterioration (yes/no)	−2.600	0.505	26.457	1	0.000	0.074
Pandemic-related worry levels of carer (+: higher worry)	−0.648	0.234	7.686	1	0.006	0.523
A deterioration in the emotional regulation capabilities of the care receiver (yes/no)	−1.214	0.475	6.536	1	0.011	0.297
Abandonment is mentioned among problems (yes/no)	−1.187	0.535	4.918	1	0.027	0.305
Constant	4.051	1.081	14.051	1	0.000	57.430
*Hungarian sample*						
Carer’s general health deterioration (yes/no)	−2.703	0.552	23.940	1	0.000	0.067
Pandemic-related worry levels of carer (+: higher worry)	−1.063	0.310	11.757	1	0.001	0.346
Decline in the carer–care receiver relationship (yes/no)	−1.134	0.483	5.519	1	0.019	0.322
Carer had care-related help from the family doctor during the first wave of the pandemic (yes/no)	1.534	0.725	4.483	1	0.034	4.637
Constant	4.143	1.233	11.297	1	0.001	63.001

**Table 4:** Variables with significant partial predictive power over carers’ high pandemic-related worry levels (conditional forward stepwise method, final model).

	B	S.E.	Wald	df	Sig.	Exp(B)
*Italian sample*						
Being the child of care receiver (yes/no)	−1.762	0.458	14.765	1	0.000	0.172
Having lost the help the carer had for daytime surveillance before the pandemic (yes/no)	1.423	0.491	8.387	1	0.004	4.151
Carer’s general health deterioration (yes/no)	−1.275	0.472	7.308	1	0.007	0.280
Anxiety is mentioned among problems (yes/no)	−1.378	0.674	4.182	1	0.041	0.252
Constant	0.422	0.412	1.048	1	0.306	1.525
*Hungarian sample*						
Physical deterioration of the care receiver (yes/no)	−2.424	0.645	14.131	1	0.000	0.089
Feeling highly time-constrained between tasks (yes/no)	−0.623	0.180	11.908	1	0.001	0.536
Increase in the carer’s care time (yes/no)	1.139	0.447	6.494	1	0.011	3.122
Conversation, communication is among the carer’s tasks (yes/no)	1.662	0.736	5.096	1	0.024	5.272
Constant	2.587	0.742	12.152	1	0.000	13.291

**Table 5:** Variables with significant partial predictive power for feeling highly overwhelmed (conditional forward stepwise method, final model.

	B	S.E.	Wald	df	Sig.	Exp(B)
*Italian sample*						
Pandemic-related worry levels of carer (+: higher worry)	−0.680	0.262	6.725	1	0.010	0.506
Decline in the carer–care receiver relationship (yes/no)	−1.056	0.393	7.235	1	0.007	0.348
Having had some kind of care-related help at all during the first wave of the pandemic (yes/no)	−1.019	0.399	6.522	1	0.011	0.361
Having moved in together with the care receiver during the first wave of the pandemic (yes/no)	−1.210	0.621	3.795	1	0.051	0.298
Constant	4.239	1.199	12.501	1	0.000	69.367
*Hungarian sample*						
Pandemic-related worry levels of carer (+: higher worry)	−0.912	0.208	19.199	1	0.000	0.402
Carer had care-related help from social service providers during the first wave of the pandemic (yes/no)	−1.228	0.570	4.641	1	0.031	0.293
Being the child of care receiver (yes/no)	−0.856	0.370	5.355	1	0.021	0.425
Jobs around the house are among the carer’s tasks (yes/no)	0.714	0.355	4.049	1	0.044	2.042
Constant	4.491	1.052	18.226	1	0.000	89.195

## Data Availability

Data is not yet publicly available, but is planned to be made available at the end of 2022.

## Appendix A

**Table A1:** Variables in a significant relationship with the general health deterioration of carers.

		Italian Sample					Hungarian Sample				
		*n*	Experienced g. Health Deterioration	χ^2^	*p*	Phi	*n*	Experienced g. Health Deterioration	χ^2^	*p*	Phi
Carer’s mental health deteriorated	Yes	104	66%	62.043	<0.001	0.574	71	54%	46.624	<0.001	0.506
	No	84	10%				111	8%			
Carer-care receiver relationship deteriorated	Yes	70	63%	22.118	<0.001	0.343	52	42%	10.326	0.001	0.238
	No	118	28%				130	19%			
State of the care receiver deteriorated during 1st wave	Yes	115	52%	12.361	<0.001	0.272	67	34%	4.803	0.028	0.18
	No	52	23%				81	19%			
Carer mentioned abandonment (no help with care, difficulty with care tasks, isolation from patient) as a problem	Yes	45	64%	13.496	<0.001	0.268	11	27%	not sig.		
	No	143	34%				171	26%			
Carer did not get help during 1st wave despite needing it	Yes	55	56%	7.631	0.006	0.201	31	39%	not sig.		
	No	132	35%				150	23%			
Carer’s care time increased	Yes	110	49%	7.253	0.007	−0.196	106	30%	not sig.		
	No	78	29%				67	22%			
Emotional deterioration (e.g., aggression, apathy) of the care receiver occurred	Yes	68	53%	6.327	0.012	0.183	18	22%	not sig.		
	No	120	34%				164	26%			
Carer mentioned their own physical/mental deterioration (exhaustion, insomnia, health problems, frustration, stress, hopelessness, depression) as a problem	Yes	27	63%	6.313	0.012	0.183	19	37%	not sig.		
	No	161	37%				163	25%			
Shopping is among carer’s tasks	Yes	162	44%	5.89	0.015	−0.177	158	25%	not sig.		
	No	26	19%				24	29%			
Dealing with official affairs on behalf of the care receiver is among carer’s tasks	Yes	153	45%	5.827	0.016	−0.176	157	26%	not sig.		
	No	35	23%				25	24%			
Carer had help from family during 1st wave	Yes	69	30%	5.209	0.022	0.167	99	25%	not sig.		
	No	118	48%				82	27%			
Carer “gained” the help of family during 1st wave (who did not have it before)	Yes	14	14%	4.518	0.034	0.155	12	8%	not sig.		
	No	173	43%				165	27%			
Carer “lost” the help they had for everyday tasks (any of: housework, personal hygiene of the dementia patient, daytime surveillance) (those who had this type before the pandemic)	Yes	54	54%	3.807	0.051	0.155	25	16%	not sig.		
	No	104	38%				119	28%			
Physical deterioration (e.g., motor coordination) of the care receiver occurred	Yes	32	56%	not sig.			22	55%	10.777	0.001	−0.243
	No	156	38%				160	22%			
Carer is the child of the care receiver	Yes	144	40%	not sig.			125	20%	7.068	0.008	−0.197
	No	44	43%				57	39%			
Patient movement is among carer’s tasks	Yes	109	44%	not sig.			53	38%	5.539	0.019	−0.174
	No	79	37%				129	21%			
Feeding is among carer’s tasks	Yes	53	45%	not sig.			96	34%	5.308	0.021	−0.171
	No	135	39%				86	19%			
Cooking is among carer’s tasks	Yes	121	40%	not sig.			146	29%	5.072	0.024	−0.167
	No	67	42%				36	11%			
Carer did not need help during 1st wave	Yes	25	24%	not sig.			25	8%	4.871	0.027	0.164
	No	162	44%				156	29%			
Bathing is among carer’s tasks	Yes	119	44%	not sig.			109	31%	4.089	0.043	−0.150
	No	69	36%				73	18%			
		Relationship with general health change in the Italian sample					Relationship with general health change in the Hungarian sample				
		*n*		Spearman’s rho *		*p*	*n*		Spearman’s rho *		*p*
Carer’s pre-pandemic health (+: better health)		188		0.418		<0.001	182		0.533		<0.001
Number of deterioration symptoms (+: more symptoms)		188		−0.254		<0.001	182		−0.171		0.021
Carer’s worry levels (+: higher worry)		188		−0.199		0.006	182		−0.162		0.029
Carer’s agreement with the statement “I feel time-constrained among my many tasks” (+: higher agreement)		188		−0.247		0.001	182		not sig.		
Age of carer		188		not sig.			182		−0.223		0.003
Number of care tasks		188		not sig.			182		−0.212		0.004

* A positive Spearman’s rho indicates a positive relationship (an increase in the value of the variable in the first column correlates to a better general health outcome); a negative Spearman’s rho indicates the opposite.

**Table A2:** Variables in a significant relationship with the mental health deterioration of carers.

		Italian Sample					Hungarian Sample				
		*n*	Experienced m. Health Deterioration	χ^2^	*p*	Phi	*n*	Experienced m. Health Deterioration	χ^2^	*p*	Phi
Carer’s general health deteriorated	Yes	77	90%	62.043	<0.001	0.574	47	90%	46.621	<0.001	0.506
	No	111	32%				135	25%			
Carer–care receiver relationship deteriorated	Yes	70	81%	30.759	<0.001	0.404	52	67%	24.500	<0.001	0.367
	No	118	40%				130	28%			
State of the care receiver deteriorated during 1st wave	Yes	115	70%	18.040	<0.001	0.329	67	48%	5.125	0.024	0.186
	No	52	35%				81	30%			
Carer’s care time increased	Yes	110	66%	13.084	<0.001	−0.264	106	46%	5.494	0.019	−0.178
	No	78	40%				67	28%			
Carer mentioned their own physical/mental deterioration (exhaustion, insomnia, health problems, frustration, stress, hopelessness, depression) as a problem	Yes	27	74%	4.487	0.034	0.154	19	74%	10.720	0.001	0.243
	No	161	52%				163	35%			
Carer mentioned abandonment (no help with care, difficulty with care tasks, isolation from patient) as a problem	Yes	45	84%	20.304	<0.001	0.329	11	45%	not sig.		
	No	143	46%				171	39%			
Emotional deterioration (e.g., aggression, apathy) of the care receiver occurred	Yes	68	76%	19.283	<0.001	−0.320	18	44%	not sig.		
	No	120	43%				164	38%			
Carer did not get help during 1st wave despite needing it	Yes	55	73%	9.243	0.002	−0.222	31	42%	not sig.		
	No	132	48%				150	39%			
Carer did not need help during 1st wave	Yes	25	28%	8.915	0.003	0.218	23	22%	not sig.		
	No	162	60%				156	41%			
Carer used to get help before the pandemic but did not get help during the 1st wave	Yes	37	76%	6.902	0.009	0.209	12	67%	not sig.		
	No	121	51%				132	36%			
Physical deterioration (e.g., motor coordination) of the care receiver occurred	Yes	32	63%	not sig.			22	73%	11.957	0.001	−0.256
	No	156	54%				160	34%			
Carer had help from healthcare providers (any of: family doctor, specialist, medical assistant, ambulance) during 1st wave	Yes	40	60%	not sig.			37	62%	10.262	0.001	−0.238
	No	146	54%				144	33%			
Carer had help from family doctor during 1st wave	Yes	30	53%	not sig.			28	64%	8.725	0.003	−0.220
	No	157	56%				153	35%			
Carer had non-family voluntary help (any of: charities, church, colleagues, neighbours, friends, volunteers, telephone helpline) during 1st wave	Yes	22	68%	not sig.			25	64%	7.467	0.006	−0.203
	No	164	54%				156	35%			
Carer stayed in full-time employment	Yes	31	58%	not sig.			32	19%	6.699	0.01	0.192
	No	157	55%				150	43%			
Patient movement is among carer’s tasks	Yes	109	54%	not sig.			53	53%	6.002	0.014	−0.182
	No	79	57%				129	33%			
Carer experienced financial difficulties during 1st wave	Yes	44	57%	not sig.			49	53%	5.394	0.02	−0.173
	No	130	54%				132	34%			
Feeding is among carer’s tasks	Yes	53	53%	not sig.			86	47%	3.855	0.05	−0.146
	No	135	56%				96	32%			
		Relationship with mental health change in the Italian sample					Relationship with mental health change in the Hungarian sample				
		*n*		Spearman’s rho *		*p*	*n*		Spearman’s rho *		*p*
Carer’s pre-pandemic health		188		0.262		<0.001	182		0.205		0.006
Number of deterioration symptoms (+: more symptoms)		188		−0.250		0.001	182		−0.212		0.004
Carer’s worry levels (+: higher worry)		188		−0.223		0.002	182		−0.344		<0.001
Number of care tasks		188		not sig.			182		−0.179		0.016

* A positive Spearman’s rho indicates a positive relationship (an increase in the value of the variable in the first column correlates to a better mental health outcome); a negative Spearman’s rho indicates the opposite.

**Table A3:** Variables in a significant relationship with carers’ high worry levels.

		Italian Sample					Hungarian Sample				
		*n*	Has High Worry Levels	χ^2^	*p*	Phi	*n*	Has High Worry Levels	χ^2^	*p*	Phi
Carer is the child of the care receiver	Yes	144	80%	11.208	0.001	0.244	125	48%	4.311	0.038	0.154
	No	44	55%				57	32%			
Carer is the partner of the care receiver	Yes	30	50%	10.613	0.001	−0.238	32	31%	not sig.		
	No	158	78%				150	45%			
Carer “lost” the help received with daytime surveillance (those who had it before the pandemic)	Yes	32	56%	7.847	0.005	−0.223	19	42%	not sig.		
	No	126	80%				125	42%			
Carer’s health deteriorated	Yes	77	86%	9.388	0.002	0.223	47	53%	not sig.		
	No	111	66%				135	39%			
Carer mentioned anxiety (general, worry, fear of infection) among problems	Yes	42	90%	7.678	0.006	0.202	15	47%	not sig.		
	No	146	69%				167	43%			
Carer is retired	Yes	28	54%	7.080	0.008	0.194	58	34%	not sig.		
	No	160	78%				124	47%			
Carer stayed in full-time employment	Yes	31	90%	5.172	0.023	−0.166	32	53%	not sig.		
	No	157	71%				150	41%			
Carer changed to working from home during 1st wave	Yes	50	86%	5.144	0.023	−0.165	36	47%	not sig.		
	No	138	70%				146	42%			
Carer did not get help during 1st wave despite needing it	Yes	55	84%	3.796	0.051	0.142	29	41%	not sig.		
	No	133	70%				150	44%			
State of the care receiver deteriorated during 1st wave	Yes	115	73%	not sig.			67	55%	14.435	<0.001	0.312
	No	52	75%				81	25%			
Physical deterioration (e.g., motor coordination) of the care receiver occurred	Yes	32	72%	not sig.			22	82%	15.511	<0.001	0.292
	No	156	74%				160	38%			
Carer’s care time increased during 1st wave	Yes	110	75%	not sig.			106	53%	8.801	0.003	−0.226
	No	78	72%				67	30%			
Carer–care receiver relationship deteriorated	Yes	70	81%	not sig.			52	58%	6.542	0.011	0.190
	No	118	69%				130	37%			
Carer mentions time management (clash with work, family commitments or housework, disrupted routines) among problems	Yes	27	74%	not sig.			18	67%	4.624	0.032	0.159
	No	161	74%				164	40%			
Conversation, communication is among carer’s tasks	Yes	127	76%	not sig.			163	45%	4.119	0.042	−0.150
	No	61	70%				19	21%			
Carer mentioned patient’s quality of life (difficulty keeping them occupied or making them understand pandemic, no social life or exercise for them, dealing with their emotions and mental health deterioration) among problems	Yes	34	71%	not sig.			13	69%	3.976	0.046	0.148
	No	154	75%				169	41%			
		Relationship with carers’ high worry levels in the Italian sample					Relationship with carers’ high worry levels in the Hungarian sample				
		*n*		Spearman’s rho *		*p*	*n*		Spearman’s rho *		*p*
Carer’s agreement with the statement “I feel time constrained among my many tasks” (+: higher agreement)		188		0.236		0.001	182		0.339		<0.001
Age of carer		188		−0.178		0.015	182		not sig.		
Number of deterioration symptoms		188		not sig.			182		0.240		0.001

* A positive Spearman’s rho indicates a positive relationship (an increase in the value of the variable in the first column correlates to a lower worry level outcome); a negative Spearman’s rho indicates the opposite.

**Table A4:** Variables in a significant relationship with carers’ high subjective overwhelmedness.

		Italian Sample					Hungarian Sample				
		*n*	Feels Highly Overwhelmed	χ2	*p*	Phi	*n*	Feels Highly Overwhelmed	χ2	*p*	Phi
Carer newly moved in together with care receiver (during 1st wave)	Yes	21	76%	12.989	<0.001	0.320	13	69%	not sig.		
	No	167	35%				169	55%			
Carer–care receiver relationship deteriorated	Yes	70	56%	11.641	0.001	0.249	52	63%	not sig.		
	No	118	31%				130	53%			
Carer’s general health deteriorated	Yes	77	55%	11.676	0.001	0.249	47	53%	not sig.		
	No	111	30%				135	57%			
Carer’s care time increased during 1st wave	Yes	110	49%	9.353	0.002	−0.223	106	60%	not sig.		
	No	78	27%				67	54%			
Carer did not get help during 1st wave despite needing it	Yes	55	56%	8.795	0.003	0.216	29	55%	not sig.		
	No	133	33%				150	57%			
Deterioration in the emotion regulation of the care receiver occurred (e.g., aggression, apathy)	Yes	68	53%	7.563	0.006	0.201	18	56%	not sig.		
	No	120	33%				164	56%			
Carer “lost” all help from before the pandemic (those who did receive some help)	Yes	37	57%	4.866	0.027	0.176	12	58%	not sig.		
	No	121	36%				132	60%			
Carer “lost” the help received with the personal hygiene of the care receiver (those who had it before the pandemic)	Yes	31	58%	4.563	0.033	0.170	14	57%	not sig.		
	No	127	37%				130	60%			
Carer had help for mental health (any of: conversation, emotional support) during the 1st wave	Yes	30	23%	4.536	0.033	0.166	58	67%	not sig.		
	No	135	44%				91	54%			
Carer mentioned their own physical/mental deterioration (exhaustion, insomnia, health problems, frustration, stress, hopelessness, depression) as a problem	Yes	27	59%	4.931	0.026	0.162	19	68%	not sig.		
	No	161	37%				163	55%			
Carer is inexperienced (has been caring for care receiver for 1 year or less)	Yes	17	65%	4.799	0.028	0.160	35	57%	not sig.		
	No	171	37%				147	56%			
Carer experienced financial difficulties during 1st wave	Yes	44	52%	3.918	0.048	−0.150	49	63%	not sig.		
	No	130	35%				132	54%			
Carer had help from family doctor during 1st wave	Yes	30	23%	4.185	0.041	0.150	28	61%	not sig.		
	No	157	43%				153	55%			
Carer mentions time management (clash with work, family commitments or housework, disrupted routines) among problems	Yes	27	41%	not sig.			18	83%	6.039	0.014	0.182
	No	161	40%				164	53%			
Carer is the child of care receiver	Yes	144	41%	not sig.			125	62%	5.001	0.025	0.166
	No	44	36%				57	44%			
Carer has jobs around the house among their tasks	Yes	94	43%	not sig.			116	62%	4.714	0.030	−0.161
	No	94	37%				66	45%			
Carer “lost” the help of social service providers (those who had this help before the pandemic)	Yes	12	50%	not sig.			19	79%	4.574	0.032	0.161
	No	175	39%				158	53%			
Carer had help from social service providers during 1st wave	Yes	5	20%	not sig.			20	35%	3.945	0.047	0.148
	No	182	41%				161	58%			
Carer has cleaning among their tasks	Yes	128	40%	not sig.			160	59%	3.935	0.047	−0.147
	No	60	40%				22	36%			
		Relationship with carers’ high subjective overwhelmedness in the Italian sample					Relationship with carers’ high subjective overwhelmedness in the Hungarian sample				
		*n*		Spearman’s rho *		*p*	*n*		Spearman’s rho *		*p*
Carer’s worry levels (+: higher worry)		188		0.236		0.001	182		0.339		<0.001

* A positive Spearman’s rho indicates a positive relationship (an increase in the value of the variable in the first column correlates to a lower subjective overwhelmedness outcome); a negative Spearman’s rho indicates the opposite.
